# Supplementary material for: A Comprehensive Analysis of the Genomic and Expressed Repertoire of the T-Cell Receptor Beta Chain in Equus caballus
Source: Animals (Basel). 2024 Sep 29;14(19):2817. doi: 10.3390/ani14192817 (PMC11475548; doi:10.3390/ani14192817)
Supplement: Supplementary file 1 [file animals-14-02817-s001.zip › Figure S1.pdf]

(A)

Percent Identity Matrix

1: L36092|TRBV1|Homsap	100.00	47.13	45.45	45.22
2: AE000663|TRBV1|Musmus	47.13	100.00	73.84	74.86
3: TRBV1|Equcab	45.45	73.84	100.00	82.27
4: IMGT000084|TRBV1|Bostau	45.22	74.86	82.27	100.00

(B)

		FR1-IMGT (1-26)		CDR1-IMGT (27-38)	FR2-IMGT (39-55)		CDR2-IMGT (56-65)	FR3-IMGT (66-104)				CDR3-IMGT (105-117)	
		A (1-15)	B (16-26)	BC (27-38)	C (39-46)	C' (47-55)	C'C" (56-65)	C" (66-74)	D (75-84)	E (85-96)	F (97-104)	FG	
<u>TRBV1</u>	<u>Functionality</u>	1 10 15	16 23 26	27 38	39 41 46	47 55	56 65	66 74	75 80 84	85 89 96	97 104	105	
		|........|....|	|......|..|	|..........|	|.|....|	|.......|	|........|	|.......|	|....|...|	|...|......|	|......|	|......	
Equcab	F	ASFVEQKPRWVLVAH	GRAETLRCILR	NSQ......YPW	MSWYQQDL	QGQLEVLAT	LRS....SGD	EEVVSRPGA	DYRIKRV.ND	TELKLHVANVTQ	ARTLYCTC	SK.....	[6.6.2]
Musmus	F	VTLLEQNPRWRLVPR	GQAVNLRCILK	NSQ......YPW	MSWYQQDL	QKQLQWLFT	LRS....PGD	KEVKSLPGA	DYLATRV.TD	TELRLQVANMS.	.QGRTLYC	TCSA...	[6.6.4]
Bostau	F	TSLVEQKPRWVLVPR	RQAETLRCILK	DSQ......YPW	MSWYQQDL	RGQLQVLAS	LRR....TGD	KEVINLPGA	NYRATRV.SE	SELSLHVANVT.	.QGRTLFC	TCSK...	[6.6.4]
Homsap	P	DTGITQTPKYLVTAM	GSKRTMKREHL	GH........DS	MYWYRQKA	KKSLEFMFY	YNC....KEF	IENKTVP.N	HFTPECP.DS	SRLYLHVVALQQ	EDSAAYLC	TSSQ...	[4.6.4]
