# Supplementary material for: A Comprehensive Analysis of the Genomic and Expressed Repertoire of the T-Cell Receptor Beta Chain in Equus caballus
Source: Animals (Basel). 2024 Sep 29;14(19):2817. doi: 10.3390/ani14192817 (PMC11475548; doi:10.3390/ani14192817)
Supplement: Supplementary file 1 [file animals-14-02817-s001.zip › Figure S2(A) .pdf]

(A)

TRBV gene name	Functionality	L-REGION	FR1-IMGT (1-26)		CDR1-IMGT (27-38)	FR2-IMGT (39-55)		CDR2-IMGT (56-65)	FR3-IMGT (66-104)				CDR3-IMGT (105-117)	
			A (1-15)	B (16-26)	BC (27-38)	C (39-46)	C' (47-55)	C'C" (56-65)	C" (66-74)	D (75-84)	E (85-96)	F (97-104)	FG	
			1 10 15	16 23 26	27 38	39 41 46	47 55	56 65	66 74	75 80 84	85 89 96	97 104	105	
			|........|....|	|......|..|	|..........|	|.|....|	|.......|	|........|	|.......|	|....|...|	|...|......|	|......|	|......	
TRBV1	F	MVLFPVLWGWVLMAAVNAT	ASFVEQKPRWVLVAH	GRAETLRCILR	NSQ......YPW	MSWYQQDL	QGQLEVLAT	LRS....SGD	EEVVSRPGA	DYRIKRV.ND	TELKLHVANVTQ	ARTLYCTC	SK.....	[6.6.2]
TRBV2-1	P	MRCPCGRDLPPTLGDF*TQ	RT*SQPDPKIWVTEM	EEEVILRCNPI	SGH.......LY	LYCYGEIL	GQKVEFLIS	FYN....ENL	SEKSEIFRD	RFSVERP.DG	SFSNLKIQLREL	GDSAVYFC	ASSV...	[5.6.4]
TRBV2-2	F	MDSRLFCCVIFGLLGVGHM	ETGISQTPSHYIREM	GQNISLRCEPI	SSH.......FY	FYWYRQTL	EKGVEFLIS	VYN....KVP	SEKADFLKD	RFSAEMP.DG	SRFTLKIQPVQL	GDSAVYLC	ASSL...	[5.6.4]
TRBV2-3	P	MDTWLLCWVTFSLLEAGHT	EPEVSQTPSHQITEM	GQEVILRCDPV	SGH.......LN	LYW*RQIL	GQKVEFLIS	FYN....ENL	SEKSEILKD	*FSAERS.DG	SFSTLKIQLTEV	GVSAVYLC	ASSL...	[5.6.4]
TRBV3-1	F	MGSRLLCCVALCLLGADPL	DTSVSQTPEYLISQV	GNKKTLKCEQN	LNH.......DA	MYWYIQDS	KKLLKIMFS	YNN....KQP	IVNETAS.N	RFSPEAP.DK	AHLHLHINSLEP	GDSAVYFC	ASSK...	[5.6.4]
TRBV3-2	F	MGSRLLCCVALCLLGAGPL	DTAVSQTPKYLISRV	GNEKTLKCEQN	LSH.......NA	MYWYVQDS	KKLLKIMFS	YNN....KQP	IVNETAS.N	RFSPHAP.DN	AHLNLHINSLEP	SDSAVYFC	ASSQ...	[5.6.4]
TRBV3-3	F	MGSRLLCCVALCLLGAGPL	DTAVSQTPKYLISQV	GNKKTLNCEQT	LGH.......NA	MYWYKQDS	KKLLKIMFI	YYN....KER	DLNETVP.S	RFSPEAP.DK	AHLNLHVSSLEP	GDSAVYFC	ASSQ...	[5.6.4]
TRBV4-5	P	MS*RLLCCVALCLLREVLM	DTGITQIAKHLVMGL	RN*KSLKCEQH	LGH.......YP	ICWYKQSV	QKPLALMLV	YNY....KEL	SENKTVS.S	RFLLECP.DS	SLSHLHVDTLEL	*DSTVYFC	ASSK...	[5.6.4]
TRBV5-1	P	MGSRFLCYVALCLLGTGPV	DSGVVQTPRHLIKAR	GQQVPLRCSPI	SGH.......SR	VSWYQQAL	GQGPQFLIQ	YYM....GRE	NGKGDIL.D	*FSGRQF.SD	YSSELNMSVLEL	TDSALYL*	ASSA...	[5.6.4]
TRBV5-2	F	MGSRLLCCVALCLLGAGPV	DSGVIQTPRHLIKAK	GQQMTLRCSPI	STH.......PY	VYWYQQVL	GQGPRFLIQ	YFD....EKE	NDRGDSS.D	RFSGKQF.SN	YSSELKVSVLEL	VDSALYLC	ASSL...	[5.6.4]
TRBV5-4	ORF	MGSRLLCCVALCLLGASAV	DSGVTQTPRHLIKTK	GQQVTLRCSPI	SGH.......PR	VSWYQQAL	GQGPQFLFQ	YYM....GRE	NGKGDIP.D	RFKGQQF.SN	YCSEL-------	TDSALYLC	ASSA...	[5.6.4]
TRBV5-7	F	MSSTLLCCVALCLLGAGPV	DSGVTQTPRHLVKTR	GQQVTLRCSPM	SGH.......DT	VSWYRQPL	GQGLQFLIQ	YYR....GEE	NGKGDFP.G	RFKGQQL.DN	YSSELNVSVLEL	VDSALYFC	ASSL...	[5.6.4]
TRBV5-12	F	MGYRLLCYVALCLLGAGPV	DSGVIQTPRHVVKAK	RQQVMLKCSPI	SEH.......DT	VSWYQQAL	GQGPKFLIQ	YYN....GEV	REKGDIP.D	RFSGQQF.GN	RSSELNLTILEL	TDSALYLC	ASSL...	[5.6.4]
TRBV5-13	F	MGSRLLCCVALCLLGTGPV	DSGVTQTPRHLIKAR	GQQVTLRCSPI	SGH.......NR	VFWYQQPL	GQGPQFLFY	YYN....GKE	NEKGDVP.A	RFSGQQF.SD	YRSELNVSVLEL	KDSALYLC	ASSS...	[5.6.4]
TRBV5-15	F	MASRLLCCVALCVLGTGPV	DSGVTQTPRHLIKAR	GQQVTMRCSPI	SGH.......KS	VFWYQQPL	GQGPQFLVQ	YYN....EKE	NEKADTA.N	RFLGKQF.SN	YSSELNVSFLEL	TDSALYLC	ASSV...	[5.6.4]
TRBV5-17	P	TGSRLLCRAMLCLLGGGPV	EAGVTETPRYLIKTT	GQAVTLRCSTV	SKH.......SG	VSWYQQAL	SQDPHLLFE	FYE....NVQ	RAKGEIP.N	RFSAQQL.RD	YRSELNVSVLEL	MDSALYLC	ASSV...	[5.6.4]
TRBV5-19	F	MGSRLLCCVALCLLGAGPV	DSGVTQTPKHLIKAK	GQQMTLRCSPI	STH.......LS	VYWYQQAL	GQGPQFLIQ	YYD....GEE	REKGNIS.G	RFSGQQF.RD	RSSELNVSILEL	TDSALYLC	ASSL...	[5.6.4]
TRBV6-1	F	MSGGLLCWVAFCLLQAGAV	NAGVTQTPKFHILET	GQNTTLRCAQD	MNH.......DS	MYWYRQDL	GHGLRLIRY	SAG....AGV	TEKRDVP.D	GYRVSRS.NT	ENFSLTLESATP	SQTSVYFC	ASS*...	[5.6.4]
TRBV6-2	F	MSGGLLCWVAFCLLQAAPV	NAGVTQNPKFQVLGT	GQNMTLRCAQD	MNH.......SY	MYWYRQDL	RHGLRLIHY	SRA....ANV	TEKGDIP.E	GYRVSRP.NT	ENFLFTVESATP	SQTSVYFC	ASSY...	[5.6.4]
TRBV6-3	F	MSGGLLCWVVFCLLQAAPV	NAGVTQTPRFQVLRR	GENTTLRCTQD	MNH.......NS	MYWYRQDL	GHGLRLIHF	SVG....ADA	TANRDVP.D	GYRVSRS.DT	KNFALMLESATP	SQTSVYFC	ASS*...	[5.6.4]
TRBV6-4	F	MSNGLLCWVAFCLLQAAPV	NAGVTQTPRFQILGT	GQNTTLRCAQD	MNH.......NS	MYWYRQDL	GHGLRLIRY	SAG....AGV	TEE.DGP.D	GYRVSRS.DT	KNFSLTLVSATP	SQTSVYFC	ASSY...	[5.6.4]
TRBV6-5	F	MSGGLLCWAAFCLLQAGAV	NAGVTQTPKFHILET	GQNTTLICAQD	MNH.......DY	MYWYRQDL	GHGLRLIHY	SRT....SGV	TEARDIP.D	GYRVSRS.NT	ENFLLTLVSPTP	SKTSVYFC	ASSY...	[5.6.4]
TRBV6-6	F	MSGGLLCWVAFCLLQAGAV	NAGVIQTPKFQVLKT	GQNTTLRCTQD	MNH.......DS	TYWYRQDL	GHGLRLIHY	SVG....AGV	TANRDVP.D	GYRVSRS.NT	ENFPLILVSATP	SQTSVYFC	ASS*...	[5.6.4]
TRBV7-2	F	MGTRLLSFMTFCLLGADHT	DAGISQSPRHRVTER	GQDAALKCDPI	SGH.......TY	LYWYRQTL	GQNLEFLTY	FQD....DLA	SDTSGMPND	RYSAKRP.EG	SSSTLKIQHAEQ	GDSALYLC	ASSS...	[5.6.4]
TRBV7-3	P	MEHICVLSCVTLCLLGAGHT	DAGVPQSPRHRVTER	VQAVALGCDPI	CGG.......AT	LYWYQQTL	RQGPEFLIY	FMN....KNV	PDIPGTPNI	PFSPERP.EG	CSSTLKIQPAEQ	GD*IVYLC	ASSS...	[5.6.4]
TRBV7-7	F	MGTRLLCCLTLCLLGAGHT	DTGVSQSPRHRVTKY	GEDVAFRCDPI	SEH.......TR	LYWYRQTP	GQNMEFLTY	FQN....DFA	SDTSGMPSD	RFSAERP.KG	SYSTLKIQRAEQ	RDSAVYLC	ASSL...	[5.6.4]
TRBV7-9	F	MGTRLLCCLTFCLLGTDHT	KAGVCQSPRNRVSER	GQDVAFKCDPI	SEH.......TQ	LCWYRETL	GKGLEFLMY	FQD....DFA	SDTSGMLKD	QFSAERP.EG	SSPILKIQHTEQ	GDSAVYLC	QQLS...	[5.6.4]
TRBV7-11	F	MGTRILCWVTLCLLGADHT	DAGVSQSPRHRVTER	GQDVAFKCDPI	SGH.......TH	LYWYRQTR	GQGLEFLMY	FRD....DFA	SDTSGMLKD	WFSAERP.EG	SSSTLKIQHAEQ	GDSAVYLC	QQLS...	[5.6.4]
TRBV8-3	P	VSLNFFCVTLCLLGAGPM	DAGITQKPRYHITQT	GDKMILECSQT	MSH.......LA	MFWYRQDP	GQGPRLIHY	SGD....VNS	TAKGDIT.E	GYSVSRN.KK	EVFLLTLESATT	KQTSLYLC	ASSK...	[5.6.4]
TRBV8-4	ORF	MSLNFFCVTLCLLGAGPM	DAGITQKPRYHITQT	GGKTILECSQN	MSH.......FA	MFWYRQDP	GQGPRLIHY	SSG....VNS	TAKGDIT.E	GYSVSRK.KI	EDFPLTLESAST	KQTSLYLC	ASSK...	[5.6.4]
TRBV8-5	ORF	MSLNFFCVTLCLLGAGPM	DAGITQKPRYHITQT	GDKMILECSQT	MSH.......FA	MFWYRQDP	GQGPRLIHY	SSG....VNS	TAKGDIT.E	GYSVSRK.KI	EDFPLTLESVST	KQTSLYLC	ASSE...	[5.6.4]
TRBV8-7	P	MSLNFFCVTLCLLGAGPM	DAEITQTPR*HITQT	GDKMILECSQN	MSH.......LS	MFWYQQDP	GEGPRLIHY	STD....VRS	TTRGNVP.E	GYSVFRN.KK	ENFPLTLESAST	NQTSLYPC	ASSE...	[5.6.4]
TRBV10-1	F	MGTRLFCCVALCFLWTGQVD	AGAGITQSPHKITAT	GKRVTLTCHQT	QKH.......DY	MYWYRQDL	GHGLKLIHY	SYD....ILK	TEEGEVP.D	GYNVSRS.NI	EDFPLTLESATP	SQTSVYFC	ASSY...	[5.6.4]
TRBV10-2	P	MYTRLFCCVALCFLWTGQVD	AGAGITQSPHKITGT	GKMVTLTCHQT	QNH.......DY	MCWY*QDR	GHGLRLIHY	SNG....IGS	TDKGEVH.D	GYSVSRS.ST	ENFPLTLESATP	SQTSVYFC	ASSY...	[5.6.4]
TRBV10-3	P	MGIGLFCCVALCFLWTGQVD	AGAGITQSPHKITGT	GKRVTLRCHQT	QNH.......DY	MY*YQQDL	GHGLRLIHY	SNG....IDS	TDKGEVP.D	GYRVSRS.NT	ENFPLTLESATP	SQTSVYFC	ASSY...	[5.6.4]
TRBV11-4	F	MGTRFLCWVTICLLGAELT	EGGVTQSPRHMITGK	GQAVALWCDPI	SGH.......RA	LYWYQQTL	GQGLGLLIS	FED....EAV	ADDSQLPKH	RFSAERS.KG	GDSPLQIKPAEL	GDSAMYFC	ASSL...	[5.6.4]
TRBV12-1	ORF	MGTWALCCVLLCLLGVEPI	DAGVNQTPRHQVTKM	GQAVTLRREPI	SSH.......TA	LFWYRQTS	VQGLEFLFY	FHD....QIA	IDQKGRSKD	RFSAEMP.NR	SLSTLKIQCTEP	RDSAVYLC	ASSL...	[5.6.4]
TRBV12-2	F	MATRLLCCVALCLLGVEPT	DAGVIQTPRHQLTKM	GQTVTLTCEPI	STH.......DT	VFWYRQTS	VKGLEFLIY	FRD....QAP	MDKTGMPND	RFSAEMP.NG	SFSTLKIQRTEP	GDAAVYLC	ASSL...	[5.6.4]
TRBV12-4	P	LATRLLCCVAFYLLGVEPT	DARVTQIPRHYVTEM	EQAVTL*CEPI	YSH.......DT	LF*YRQTE	VQ*LELLIN	SCR....RDV	MNETGMPKD	RFSAEMS.DG	SFPTLKIRHTDP	GDSAMCLC	ASSL...	[5.6.4]
TRBV12-5	F	MGTRALCCVVLCLLRGEST	DAGVTQEPRHYVTKM	RQAVTLTCEPV	SNH.......TA	LFWYRQTS	VKGLKFLIY	LRN....QAP	IAQTGMPKD	RFSAEMP.NR	SLSTLKIQRTEP	GDSAMYIC	ASSL...	[5.6.4]
TRBV12-6	P	MGTRTLCCVVLCLLGVEPT	DAGVTQEPRHQVTKV	GQTVTLRCEPI	SSH.......TA	LFWYRQTL	LQGLEVLIS	FGN....QSP	IDETGMPKD	WFSAEMP.DR	SF*TLKIQCTEP	EDSAMYLC	ASSL...	[5.6.4]
TRBV12-7	F	MDTWALCCVLLCLLGVEPI	DSGVTQEPRHQVTKM	GQDVTLRCEPI	SSH.......TD	LFWYRQTS	VQGLEFLIY	FSN....QAV	IDQTGMPKD	RFSAEMP.NR	SFSTLKIQHTEP	RDSAVYLC	ASSL...	[5.6.4]
TRBV13-3	P	MLHLVLPEYPWAVQLLCCVVLCLLGAGP-	EDAVI*SPRHLIKGK	GGKAVLICYPS	SGH.......SS	VRWFQQAL	GQSPHFLTE	YYE....KEQ	REKGNIP.D	RFSAQQF.SE	YSSELDVSSLEL	EDSALCLC	ASSS...	[5.6.4]
TRBV13-4	F	MLCLVLPESARGTQLLCCVALRLLEAGPA	DAGVIQFPKHLIKGM	GGNAVLKCYPS	SGH.......SS	VCWFQQAL	GQGPQFLIK	YYE....KEQ	GEKRNIP.D	RFSAQQF.SD	YSSELDVSSLEL	EESALYLC	ASSS...	[5.6.4]
TRBV14-1	P	MVSMLLSLVSLCLLGARHT--	SAGVTQFPSHGVIEK	KQSVTLRCDPI	SGH.......EG	LYWYRHAV	GKEIKFLEY	FLR....EDK	QDDSGMPNE	*FSAERT.GG	TFSTLKVQHAEL	EDSGVYFC	GSSQ...	[5.6.4]
TRBV14-2	F	MVSMLLNLVCLCLLGARHTVE	SAGVTQFPNHRVIEK	KQAVTLRCDPI	SGH.......EA	LYWYRRAV	GKEIKFLVY	FLG....EDK	QDDSGMPNK	QFSAERI.GG	TFSTLNIQHAEL	EDSGVYFC	ASSQ...	[5.6.4]
TRBV14-3	F	MVFMLLSLVCLCLLGARHTVE	SAGVTQFPNYRVIEK	KQAVTLRCDPI	SGH.......AS	LYWYRRAV	GKEIKFLVY	FLR....KDK	QDDSGMPNE	RFSAERT.EG	TFSTLKVQHSEL	EDSGVYFC	ASSQ...	[5.6.4]
TRBV14-4	F	MLSMLLSLVSLCLLGARHT	SAGVTQFPSHRVVEK	KQSVTLRCDPV	PGH.......QA	LFWYQRAV	GKEITFLIY	FLR....ENK	QDESGMSNK	RFSAERT.NG	TFSTLKIQPAEL	EDSGVYFC	ASSQ...	[5.6.4]
TRBV15-1	F	MDLRLLYCVALCLLRVGHM	AAMVTQKPRYQVARK	GKPVTLSCSPN	LNH.......KY	MYWNQQKP	SQAPKLLFS	YYD....KEF	NNETDTS.D	NFQPSQP.NT	SFCSLSICSPGV	GDPAVYLC	ASKR...	[5.6.4]
TRBV15-2	F	MALRLLHCVALCLLGVGHM	GAIVTQEPRYQVTRK	GKTVTLGCSQN	LSH.......EY	MYWYQQKP	SQPPKLLFY	YYD....KDF	NNETDTS.D	NFQSSRP.NT	SFCSLSIRSPGM	GDSAVYLC	ASKR...	[5.6.4]
TRBV15-3	P	MALRLLHCVALCLLGVGHM	CAMVTQNLKYQITRK	GKLVTLSCFQN	LNH.......EL	MYWYQQKP	S*APKLLFS	HYD....KEF	NNETDTS.N	NFLPSRP.NT	SFCSLSICSPGV	GDSAVYLC	ASR....	[5.6.3]
TRBV15-5	F	MALRLLHCVTLCLLGIGHM	GAMVTQNPRYQITRK	GNPVNLSCSQN	LNH.......DT	MYWYQQKP	SQAPKLLFY	YYD....KEF	NNETDTS.N	NFQSSRP.NT	SFCSLSIRSPGV	GDSAVYLC	ASR....	[5.6.3]

TRBV16-4	F	MCSIFICFMVLS-LLGAGAL	DAEVTQTPGHLVEGK	EQKVKMYCVPK	KGH.......SY	VFWYQQNL	AKKFKFLIS	FQY....ENI	FGETEMPKE	RFSAECP.PN	SPCSLEIPPTEL	QDSAVYLC	ANSE...	[5.6.4]
TRBV16-5	F	MSPIFICFMVLSHLLGAGVL	DAAVTQTPGQLIKGK	EQKVKMYCVPK	KGH.......PF	IFWYQQNL	AKEFKFLIS	FQN....ENI	LEETEMPKE	RFSAECP.PN	SPCSLEIHPTEL	QDSAVYLC	ASGE...	[5.6.4]
TRBV18-3	P	MGSTLFCWVVIWLLGAGPT	NSSVIQDPRHLVRGR	GQEATSRCSLV	RAY.......SR	VYWCQRHL	*ESLKFMIY	L*K....*KV	IHNSARSAK	RFSAEFP.KE	GPSVLKIQLAEL	RDSAVYSC	ARS*...	[5.6.4]
TRBV18-6	P	MGPRLLCWVLIWLLGADPT	NAGNIQEPTNLVRER	GQEATLSCGPV	TGH.......SH	VYWY*TVL	*KGLKFMID	LQK....ENV	TYES*MPTI	HFSAEFP.KQ	GHSVFNI*LAEL	GDSAVYF*	ARS*...	[5.6.4]
TRBV20-3	F	MGDDDSRKSEKILQNKQDSGLGL	GALVSQHPSKAICMN	GTSVKIECRAM	DFQ......AQT	MFWYRQLP	KQGLTLMVA	SSDN...SSI	TYEQGFTRA	KFPINHL.NR	TFSTLTVMSVHP	ADSGLYFC	SAS....	[6.7.3]
TRBV20-7	P	MGDYDPRESDRKTSENTQDSGSGL	GVLVSQHPSRAICKN	GSSVEMECRTV	DLE......AQT	VCWYHQLP	NQGITLIAT	SIQG...SDP	TYEQGFPKV	KFPISHP.NL	TLSTLTILSAHP	ANSGLYFC	GAS....	[6.7.3]
TRBV21-1	F	MCHRLLCCVALFFWGAGSM	DTKVTQRPGHLVKGK	EQKAKMDCFPI	KTH.......IH	VYWYRRKL	EGEFEFLVY	VRN....GEI	TDKIEGFDQ	QFSVERS.QD	ERYSLEIKSTEP	GDSALYFC	ASSE...	[5.6.4]
TRBV21-2	F	MSLRLLCCVALFFWGAGSM	DTKVTQRPGHLVKGK	EQKAKMTCVPI	KAH.......IH	VYWYRRKL	EGEFEFLVY	LQN....NDV	IDKIEGFDQ	QFSAQCP.KN	ASCSLEIKSTEP	GDSALYFC	ASSE...	[5.6.4]
TRBV21-3	F	MCCRLLCCVALFFWGAGSM	DTKVTQRPGHLVKGK	EQKAKMDCVPI	KTH.......SY	VYWYRRKL	EGEFEFLVY	LQN....GKI	TDKIEGFDQ	QFSAQCP.QD	ASCSLEIKSTEP	GDSALYFC	ASSE...	[5.6.4]
TRBV21-4	F	MQCRLLCCVALFFWGAGSM	DTKVTQSPGHLVKGK	EQKAKMDCVPV	KTH.......SY	VYWYRQKL	EGEFDFLVY	LGN....GKI	TDKIEGFDQ	QFSAQCP.QD	ASCSLEIKSTEP	GDSALYFC	ASSE...	[5.6.4]
TRBV22-1	P	MGSWALCYIAFCLLGAGPV	DAKIYQMPAFLLTGA	E*DVTVECKQN	LEY.......NA	MYQYRQNP	GQGLRLIYY	SQV....VNY	VHKGDVP.E	GHAFSRE.ET	EMSPLTVRLAHI	NQTGLYLY	SG.....	[5.6.2]
TRBV22-2	ORF	MGCRALCYIAFCLLGAGPV	DAKIYQMPAFLLTGV	EQDVTLECKQN	LEY.......ND	MYQCRKDP	GQGLRLIYY	SQV....VNY	VHKGDVP.E	GHAFSRE.ET	EMSPLTVRLAHI	NQTGLHLY	SG.....	[5.6.2]
TRBV24	P	MASMLLCCVAFCLLGTGSM	DTGVTQTPRNRITKT	GKSVVPECSQT	KGH.......DQ	MYWYRQDL	GLGLQLIYY	SYD....VND	ISKGEIS.G	GYSEF*K.EK	VKFFLSLEMAVA	NQMALYFW	ASSYL..	[5.6.5]
TRBV25	F	MAVRLLWYVALYLLRAGLM	DADVFQTPKHCVTGT	GKKITLECFQS	MNH.......DN	MYWYRQDA	GRELQLLHY	SYG....VNN	TEKGDVP.S	ESTVSRL.RK	DRFSLTLESASP	SQTSLYLC	ASS*...	[5.6.4]
TRBV27-1	F	MGPWLLGYIMLCLLGAGPM	DAKVIQTPRHLITET	GKKLTVNCSQD	MNH.......DA	MYWYRQDP	GLGLKLIHY	STN....VEV	LYNGDVP.D	GYTVSRK.DK	GNFSLTLESASI	NQTSLYLC	ASSE...	[5.6.4]
TRBV27-2	F	MGPQLLGCVMLCLLGAGLM	EAKVTQAPRHLIEET	GKKLTVNCSQD	MNH.......DT	MYWYRQDP	GLGLKLIHY	SIN....EIL	-YKGDVP.D	GYAVSRK.DT	GEFPLTLESASI	NQTSLYLC	ASSE...	[5.6.4]
TRBV28-1	F	MGIRLLCVVAFCFPGLGFM	DATVTQTERYLVKRR	GDKVVMHCQQN	MDH.......NS	MFWYRQDP	ALGLQLLHF	SYR....IDD	EQKGDAP.E	GYSVSRK.KK	ENFTLILESAST	NQTSVYLC	ASSF...	[5.6.4]
TRBV28-2	F	MGFTLLCAVTFCFLGVGFM	DTNVTQTPRYLVKRI	REKVVMECSQN	MDH.......ET	MLWYRQDP	ALGLQLLHF	SIG....IDD	KQEGDAP.E	GYSVSRK.KK	ENFTLILESAST	NQTSVYLC	ASSF...	[5.6.4]
TRBV28-3	F	MGFTLLCAVAFCFLGQGFM	DATVTQTERYLVKRR	GDKVVMDCQQN	MDH.......NS	MFWYRQDP	ALGLQLLYF	SDS....VGD	KQEGDDP.E	GYSVSRK.KK	ENFTLILEPAST	NQTSVYLC	ASSL...	[5.6.4]
TRBV29	F	MLTFLLLLLELGSVF	GALLSQKPSRAICQR	GTTMKIQCEVD	TEL.......TL	MFWYSQLP	GQSLTLIAT	ANQG...SEA	TYESGFTKD	KFPISRP.TL	RFSTLTVNNTSP	EDSSFYFC	SAG...	[5.7.3]
