# Supplementary material for: A Comprehensive Analysis of the Genomic and Expressed Repertoire of the T-Cell Receptor Beta Chain in Equus caballus
Source: Animals (Basel). 2024 Sep 29;14(19):2817. doi: 10.3390/ani14192817 (PMC11475548; doi:10.3390/ani14192817)
Supplement: Supplementary file 1 [file animals-14-02817-s001.zip › Figure S2(B-D) .pdf]

(B)

TRBD gene name	5'D-NONAMER GGTTTTTGT	5'D–SPACER	5'D-HEPTAMER CACTGTG	D-REGION	3'D-HEPTAMER CACAGTG	3'D-SPACER	3'D-NONAMER ACAAAAACC
TRBD1	cgtttttgt	acaaagctgtaa	cattgtg	GGGACAGCAGGC<br>G T A G<br>G Q Q<br>D S R	cacggtg	attcaactttatggacaaccttt	acaaaaacc
TRBD2	catttttga	atcatagtgtaa	cattgtg	GGGACTGGGGGGTGGAG<br>G T G G W<br>G L G G G<br>D W G V E	cacgatg	attcaggtaaaggaggtgctttt	acaaaaagc

(C)

TRBJ gene name	Fct	J-NONAMER GGTTTTTGT	J-SPACER	J-HEPTAMER CACTGTG	J-REGION	5'splice donor
TRBJ1-1	F	atttttctc	cttgccccatgt	cactgtg	TGAACACTGAAGTCTTCTTCGGAGAAGGCACGAGGCTCACGGTTGTAG<br>**N T E V F F G E G T R L T V V**	gtaaga
TRBJ1-2	F	cattttgga	gtggccgtattc	tgatgtg	TTAACTATGACTATCACTTTGGCCCAGGAACTAAGCTGACGGTCATAG<br>**N Y D Y H F G P G T K L T V I**	gtaagc
TRBJ1-3	F	ggttttgaa	ggggatctggga	ggctgtg	CTTTGGAAACACCGTTGATTTTGGGGAGGGAAGCCGGCTCACTGTTGTAG<br>**F G N T V D F G E G S R L T V V**	gtaagc
TRBJ1-4	F	cagttttct	accaggctttac	tgttgtg	TAACTAATGAAAAACTATTTTTCGGCAATGGAACCAAGCTCTCTGTCTTGG<br>**T N E K L F F G N G T K L S V L**	gtaagt
TRBJ1-5	F	gggttttcc	acactcatgttg	tactgtg	GAGCAACCAGCCACAGCACTTTGGAGAGGGAACTCGACTCTCTGTCCTAG<br>**S N Q P Q H F G E G T R L S V L**	gtaagg
TRBJ1-6	F	ggttttagc	acagctgtctgc	agctgtg	TTCCTATAATTCGCCGCTCTACTTTGGGCCCGGCACCAGGCTCACTGTCACAG<br>**S Y N S P L Y F G P G T R L T V T**	gtatcg
TRBJ2-1	F	gaattcttg	gcagccccttcc	cactgtg	CTCTTACAATGAGCTGTTCTTCGGGCCAGGGACTCGGCTCACTGTGCTAG<br>**S Y N E L F F G P G T R L T V** L	gtaaga
TRBJ2-2	F	ggtttgctc	ctgggtccccag	ggctgtg	CGAACACGGGGCAGCTGTTCTTTGGGGACGGTTCCAAGCTGACTGTGCTCG<br>**N T G Q L F F G D G S K L T V L**	gtaagg
TRBJ2-3	F	ggtttttgt	cctggggctcgg	ggctgtg	AGTACAGACCCTCAATATTTCGGCCAGGGCACCCGGCTGACCGTGCTAG<br>**S T D P Q Y F G Q G T R L T V L**	gtaagc
TRBJ2-4	F	cgtttttgt	gctgcgcccggg	ggctgtg	AGCCAAAATACTCAGTACTTCGGCCCGGGTACTCGGCTGTCGGTGCTAG<br>**S Q N T Q Y F G P G T R L S V L**	gtaagc
TRBJ2-5	F	ggtttttgc	gcggggctcggg	ggccgtg	ACCTCAGAGACGCAGTACTTCGGGCCCGGCACGCGGCTCTTCGTGCTGG<br>**T S E T Q Y F G P G T R L F V L**	gtaagc
TRBJ2-6	F	ggttttggg	cagaagctctgg	ggctgtg	CTTTCCGGCGCCGACCAGACTTTCGGGGCCGGCAGCCGGCTGACCGTCGTGG<br>**L S G A D Q T F G A G S R L T V V**	gtgagt
TRBJ2-7	F	ggtttacgt	gtggggcttggt	ctccgtg	CTCTTACGAGCAGTACTTCGGCTCCGGCACCAAGCTCACGGTCATAG<br>**S Y E Q Y F G S G T K L T V I**	gtaaga

(D)

```
                                                          C-DOMAIN
                                                           [EX1]

                              A          AB      B         BC       C      CD       D          DE             E       EF  F             FG                     G
 TRBC                       (1-15)             (16-26)   (27-38)  (39-45)        (77-84)                     (85-96)     (97-104)         (105-117)              (118-128)
gene name     Fct         --------------->  ----------->            ------->       ------->                  ----------->  ------->                                ----------->
                         1          10   15   16     23  26 27        36 3941  45      77  80  84              85  89     96  97     104 105                 117 118121   128
                 87654321|........|....|123|......|..|  |........|  |.|...|1234567|..|...|12345677654321|...|......|12|......|  |......123456787654321.....|  |..|......|
TRBC1       F    (E)NLNQVTLPKVAVFEPSEAEISR...TEKATLVCLAT GFYP..DHVE LSWWVNGKEVQT..GVSTDPQPYREQSDD.ENPSYCLSSRLRVSAAFWH.NPRNHFRC QVQFFGLEEN..DEWKE.NRTKPVRQNI SAEAWGRA...

TRBC2       F    (E)NLNQVTLPKVAVFEPSEAEISR...TEKATLVCLAT GFYP..DHVE LSWWVNGKEVQT..GVSTDPQPYREQSDD.ENPSYCLSSRLRVSAAFWH.NPRNHFRC QVQFFGLAEN..DEWKE.NRTKPVRQNI SAEAWGRA...
```

```
                  CONNECTING-REGION        | TRANSMEMBRANE-REGION  |CYTOPLASMIC-REGION

                [EX2]            [EX3]                  [EX4]
 TRBC1      (D)CGLSS  (V)SYQRGVLSATILYEILLGKATLYAVLVSALVLMAM  VKRKDF

 TRBC2      (D)CGLSS  (V)SYQRGVLSATILYEILLGKATLYAVLVSALVLMAM  VKKKDS
```
