# Supplementary material for: A Comprehensive Analysis of the Genomic and Expressed Repertoire of the T-Cell Receptor Beta Chain in Equus caballus
Source: Animals (Basel). 2024 Sep 29;14(19):2817. doi: 10.3390/ani14192817 (PMC11475548; doi:10.3390/ani14192817)

<1 7362 MODX
Wed Jun 19 04:25:40 EDT 2024
http://pipmaker.bx.psu.edu/pipmaker/
Genome Research, Vol. 10, Issue 4, pp577-586, April 2000.

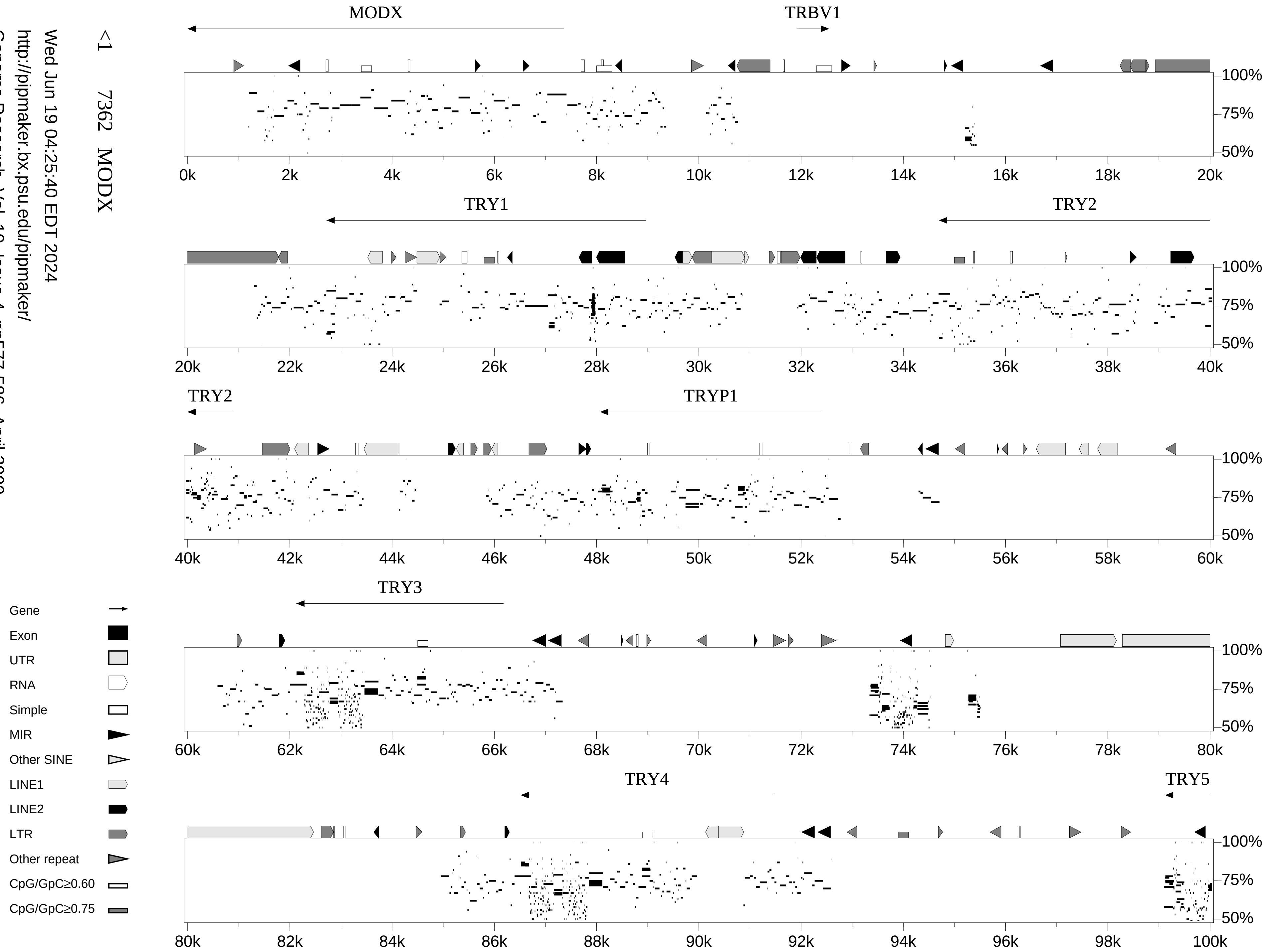

<1 7362 MODX

Wed Jun 19 04:25:40 EDT 2024

http://pipmaker.bx.psu.edu/pipmaker/

Genome Research, Vol. 10, Issue 4, pp577-586, April 2000.

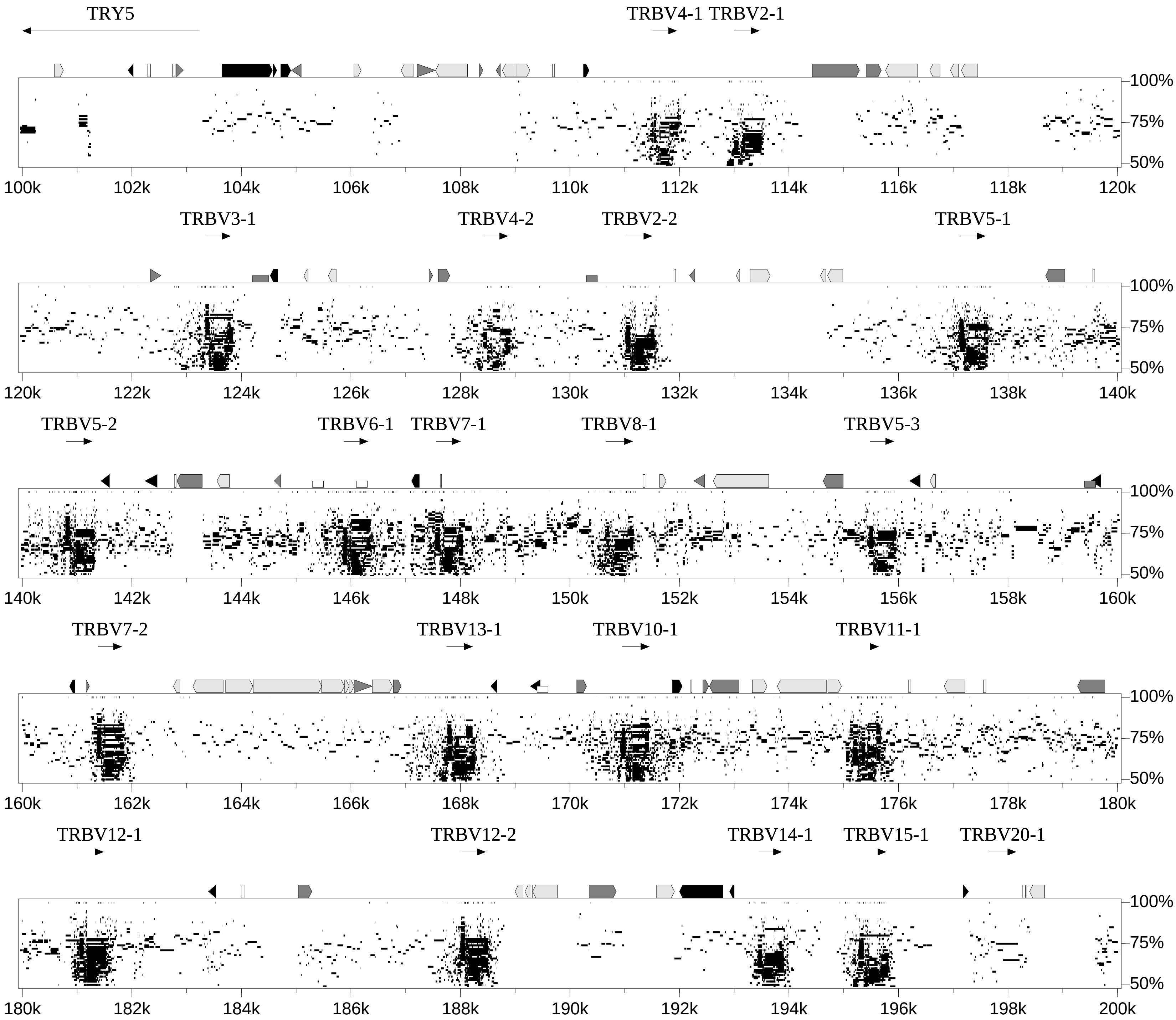

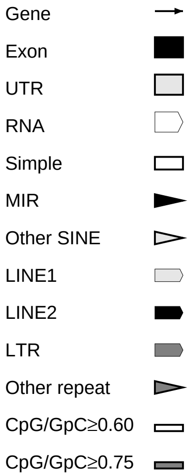

<1 7362 MODX

Wed Jun 19 04:25:40 EDT 2024

http://pipmaker.bx.psu.edu/pipmaker/

Genome Research, Vol. 10, Issue 4, pp577-586, April 2000.

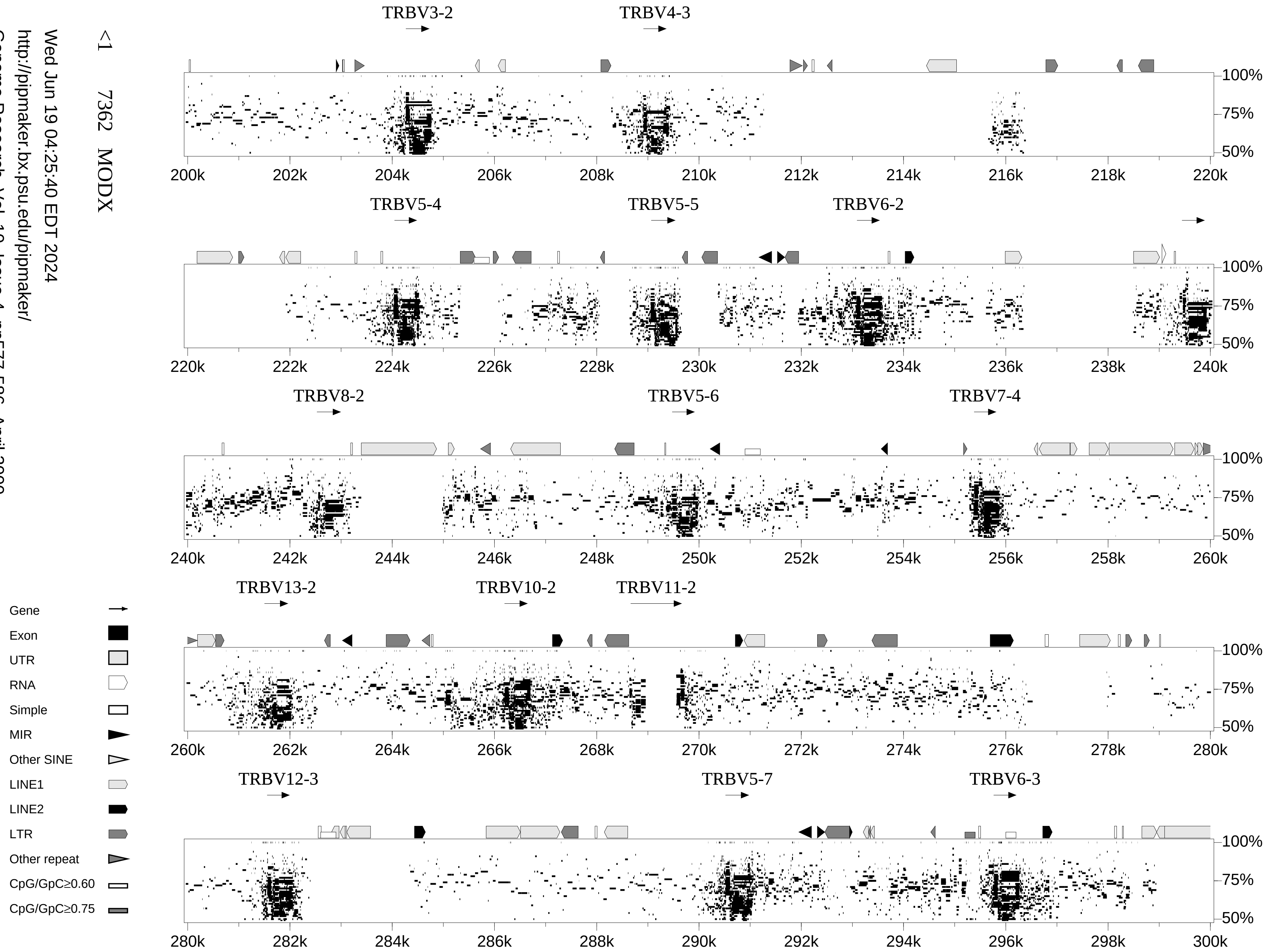

Wed Jun 19 04:25:40 EDT 2024
http://pipmaker.bx.psu.edu/pipmaker/
Genome Research, Vol. 10, Issue 4, pp577-586, April 2000.

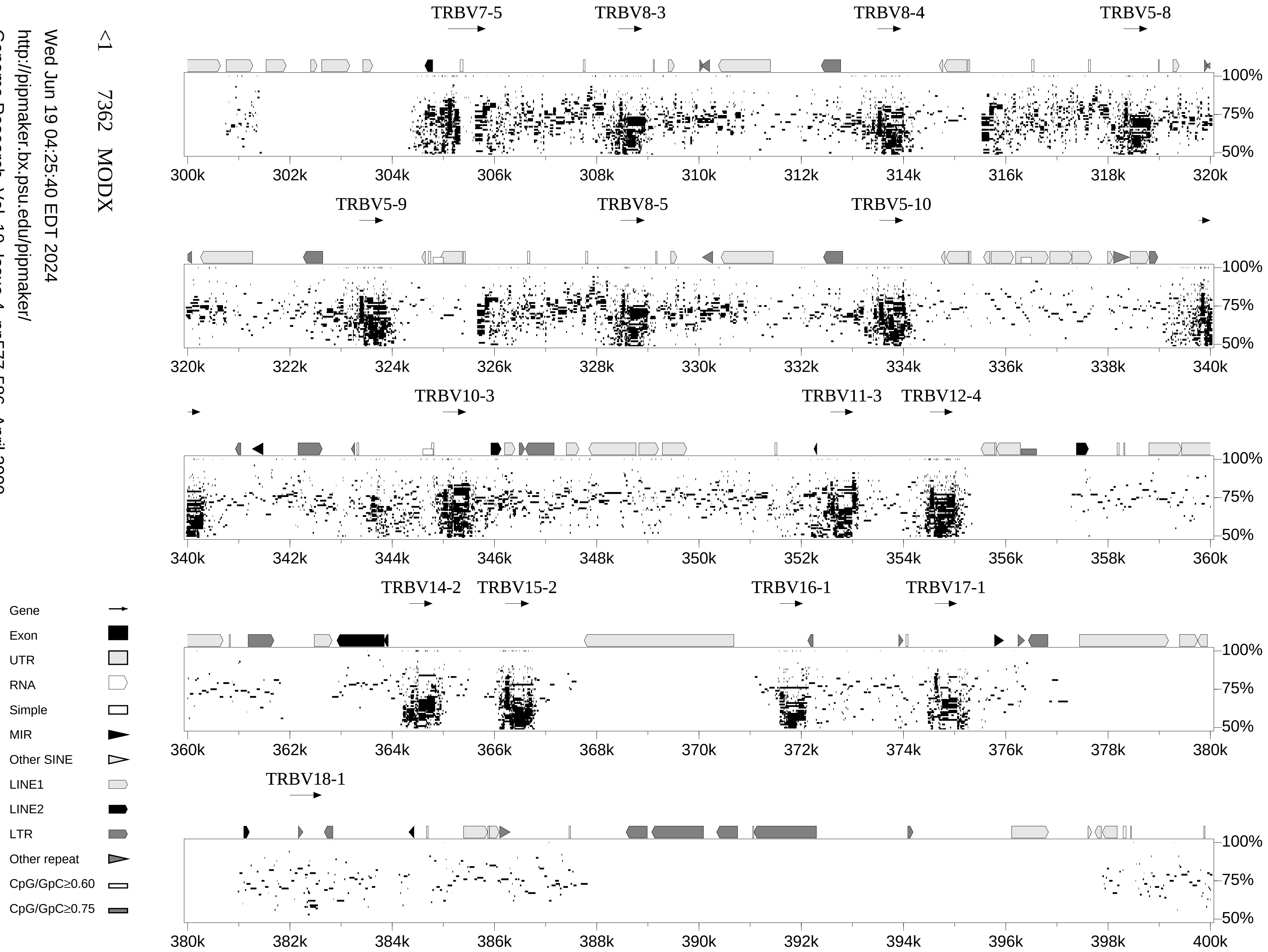

Genome Research, Vol. 10, Issue 4, pp577-586, April 2000.

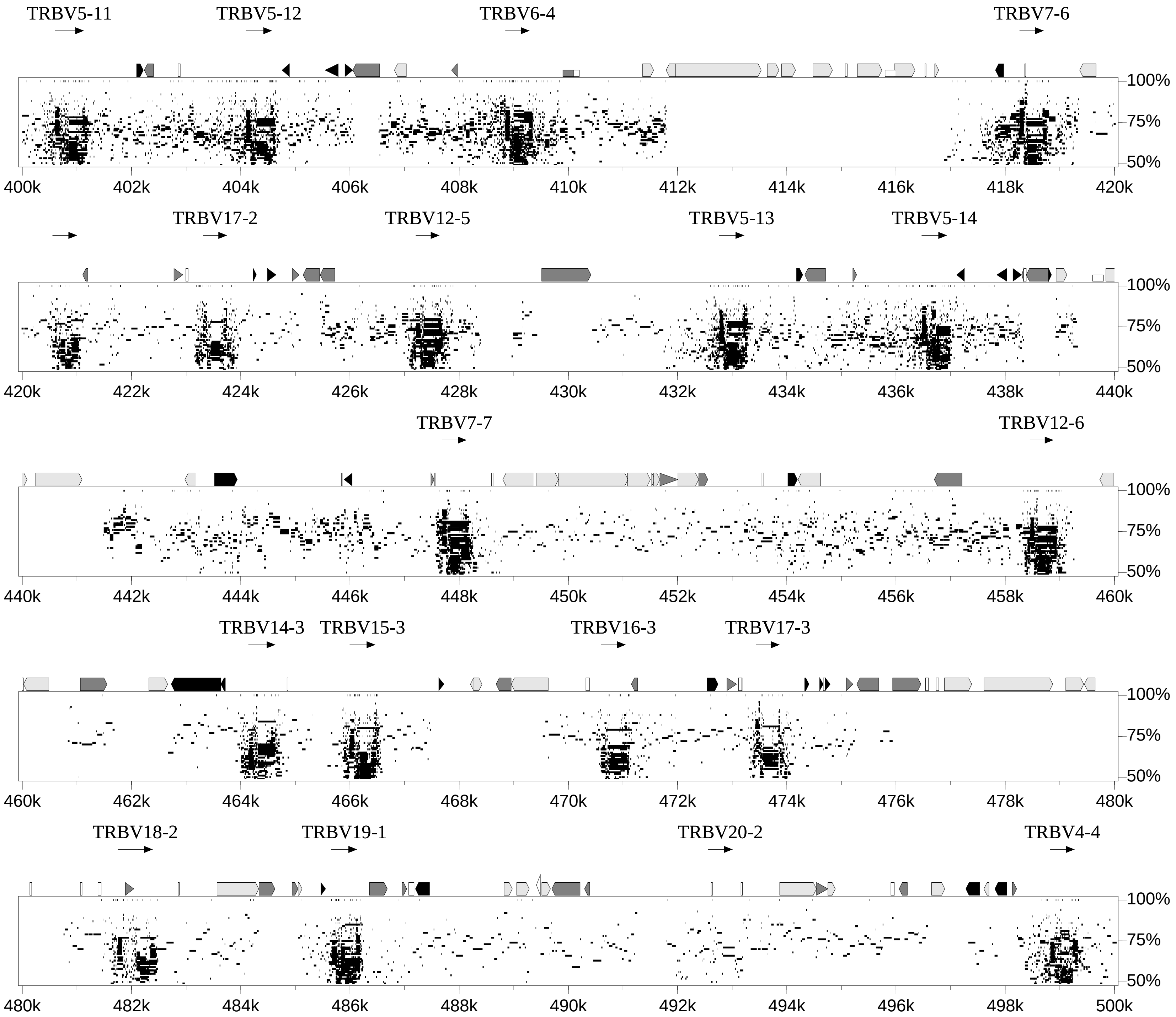

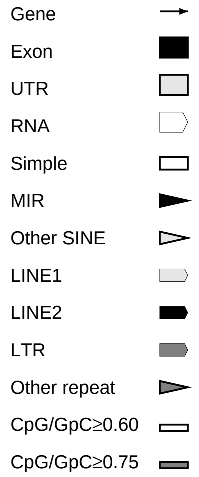

<1 7362 MODX

Wed Jun 19 04:25:40 EDT 2024

http://pipmaker.bx.psu.edu/pipmaker/

Genome Research, Vol. 10, Issue 4, pp577-586, April 2000.

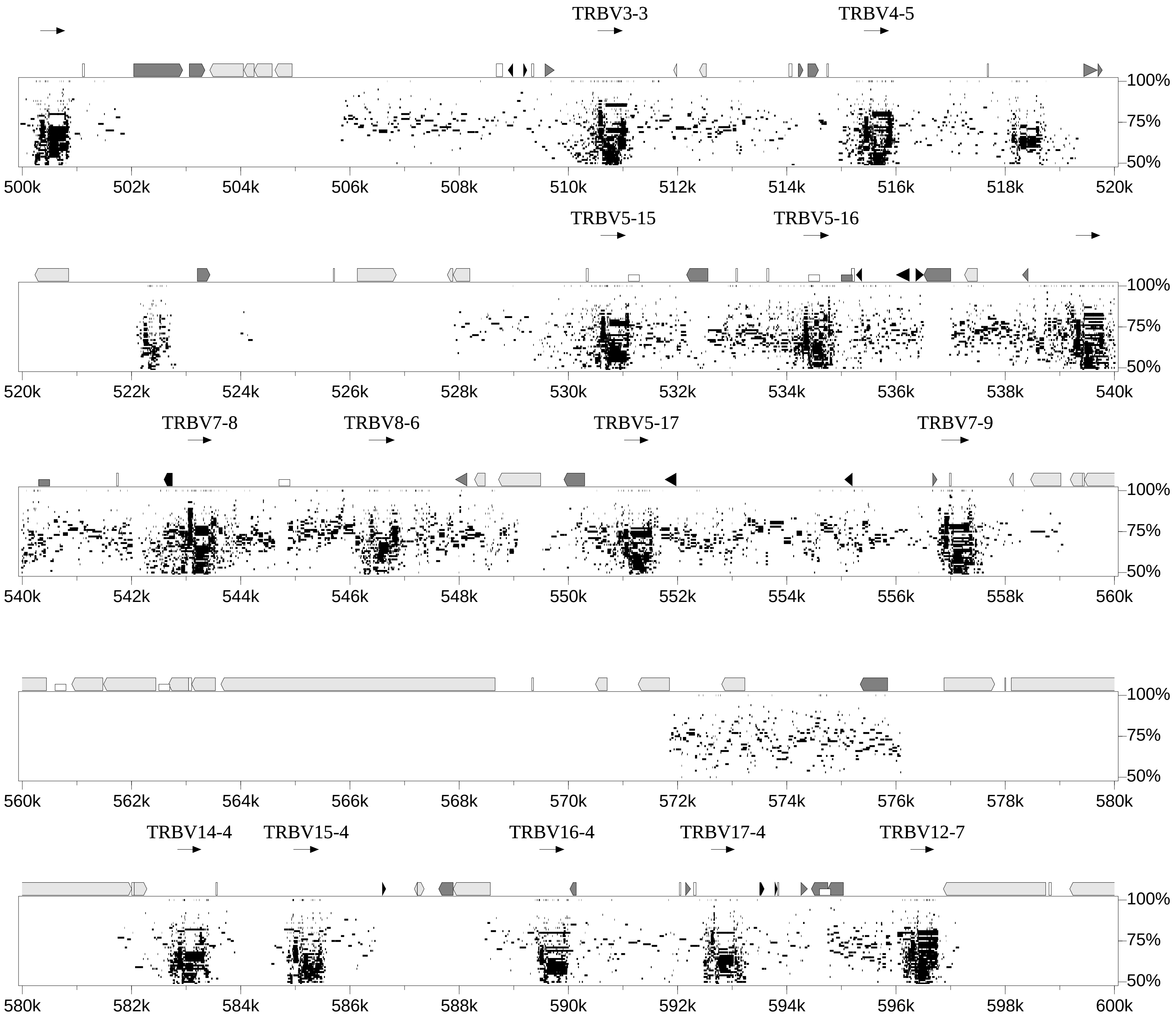

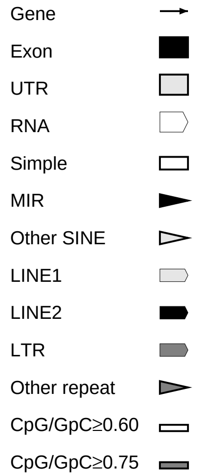

Genome Research, Vol. 10, Issue 4, pp577-586, April 2000.
http://pipmaker.bx.psu.edu/pipmaker/
Wed Jun 19 04:25:40 EDT 2024

<1 7362 MODX

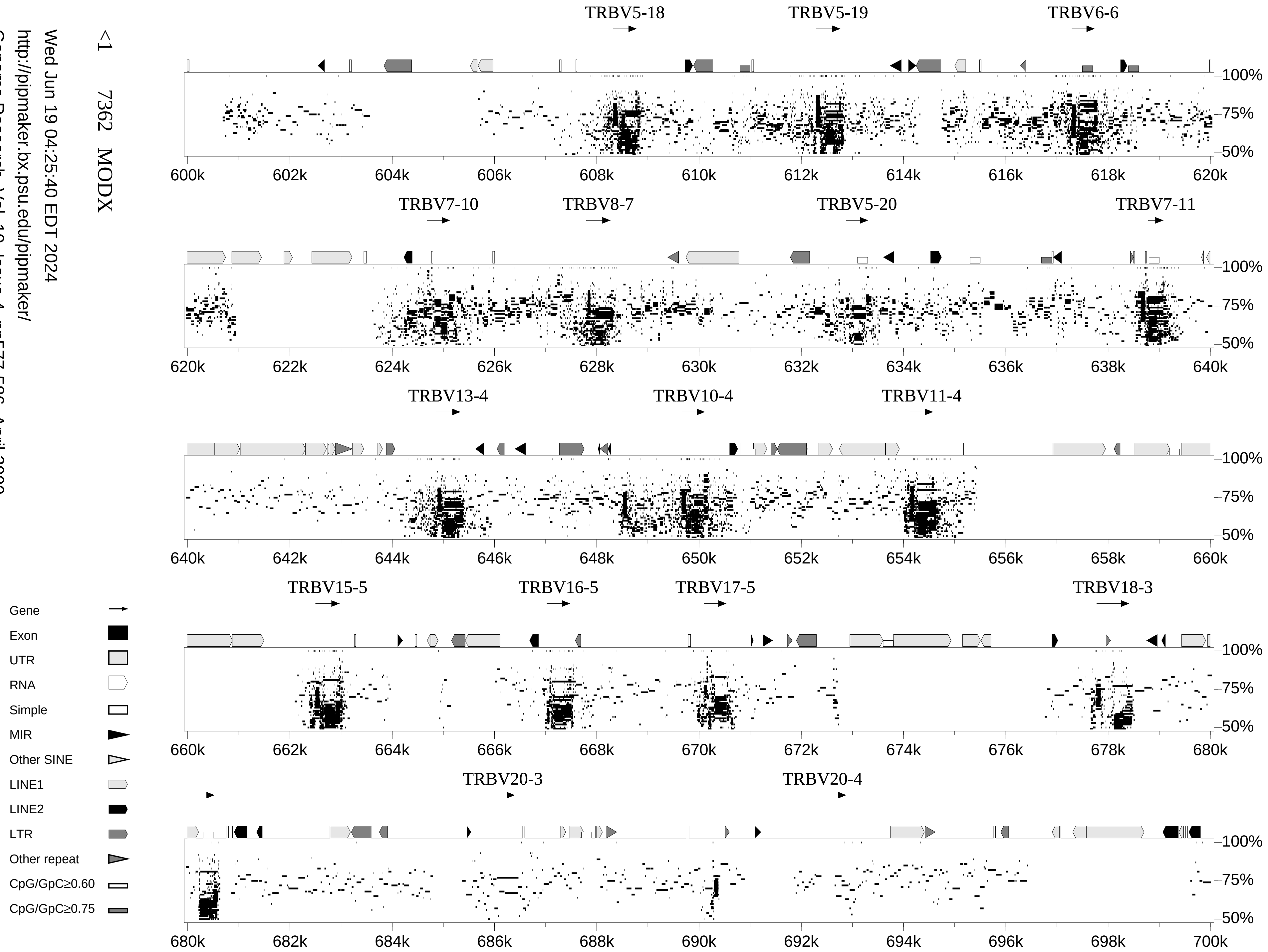

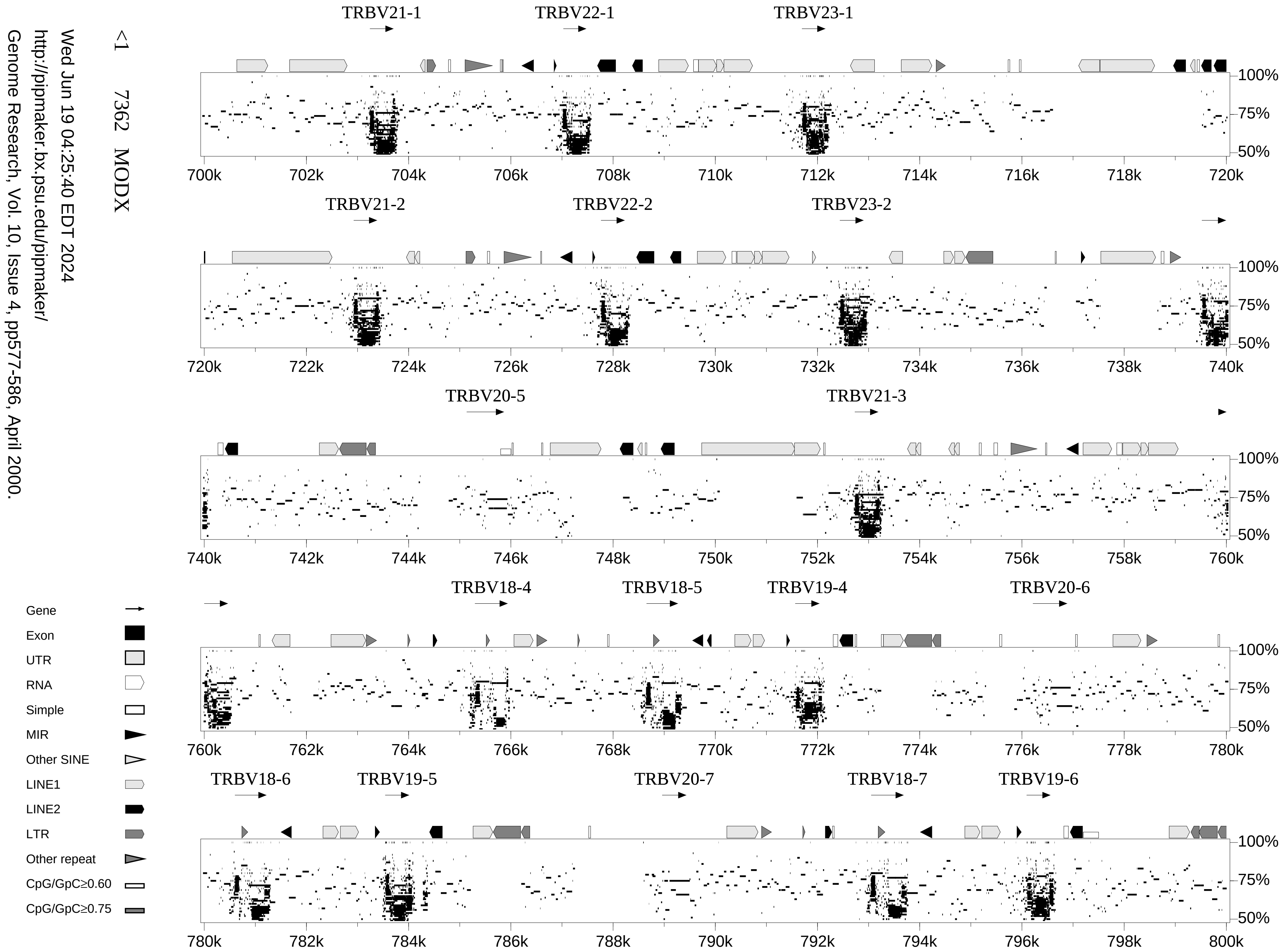

<1 7362 MODX
Wed Jun 19 04:25:40 EDT 2024
http://pipmaker.bx.psu.edu/pipmaker/
Genome Research, Vol. 10, Issue 4, pp577-586, April 2000.
TRBV21-1
TRBV22-1
TRBV23-1
700k
702k
704k
706k
708k
710k
712k
714k
716k
718k
720k
TRBV21-2
TRBV22-2
TRBV23-2
720k
722k
724k
726k
728k
730k
732k
734k
736k
738k
740k
TRBV20-5
TRBV21-3
740k
742k
744k
746k
748k
750k
752k
754k
756k
758k
760k
TRBV18-4
TRBV18-5
TRBV19-4
TRBV20-6
760k
762k
764k
766k
768k
770k
772k
774k
776k
778k
780k
TRBV18-6
TRBV19-5
TRBV20-7
TRBV18-7
TRBV19-6
780k
782k
784k
786k
788k
790k
792k
794k
796k
798k
800k
100%
75%
50%
Gene
Exon
UTR
RNA
Simple
MIR
Other SINE
LINE1
LINE2
LTR
Other repeat
CpG/GpC≥0.60
CpG/GpC≥0.75

Genome Research, Vol. 10, Issue 4, pp577-586, April 2000.
http://pipmaker.bx.psu.edu/pipmaker/
Wed Jun 19 04:25:40 EDT 2024

<1 7362 MODX

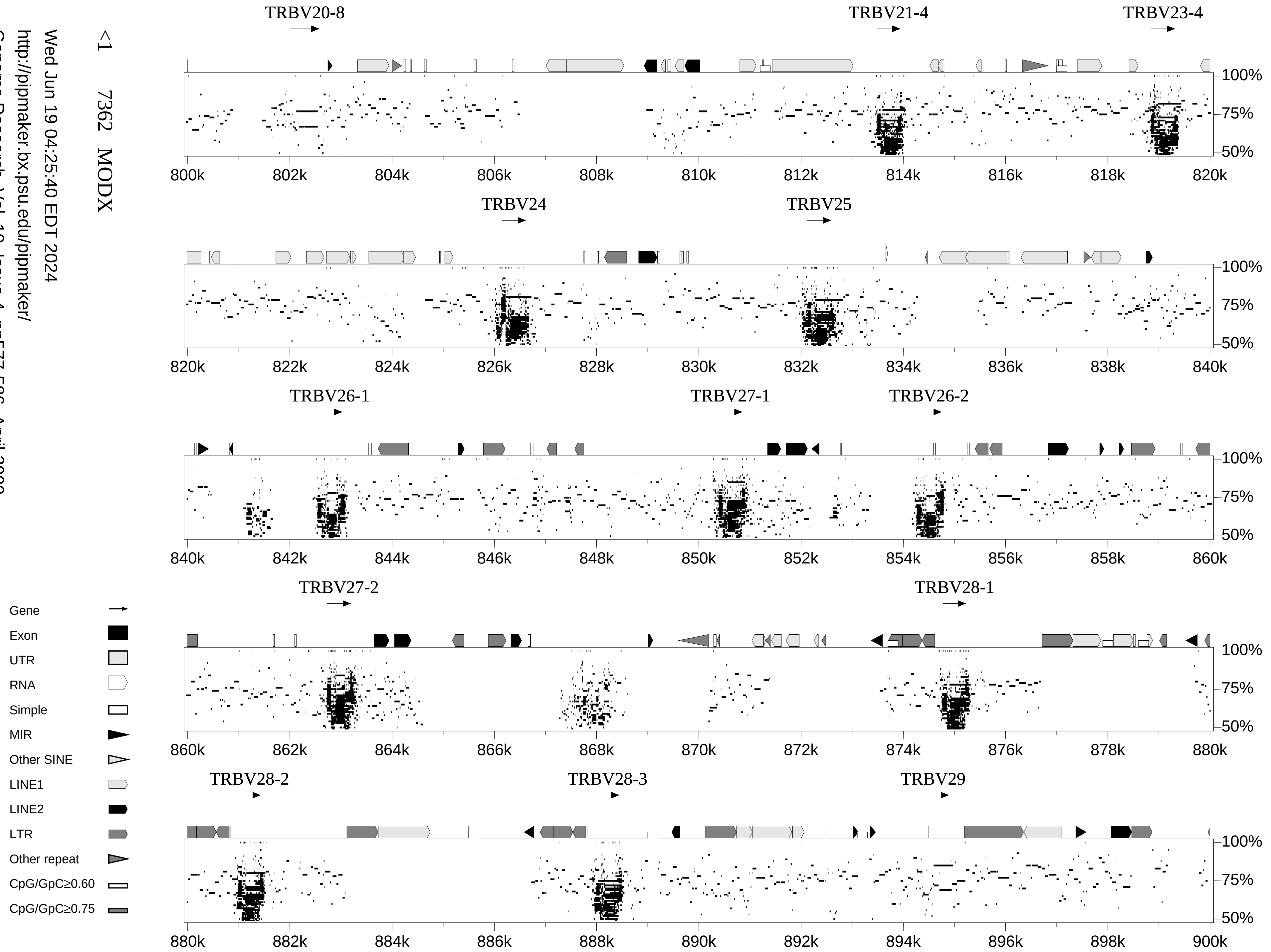

<1 7362 MODX

Wed Jun 19 04:25:40 EDT 2024

http://pipmaker.bx.psu.edu/pipmaker/

Genome Research, Vol. 10, Issue 4, pp577-586, April 2000.

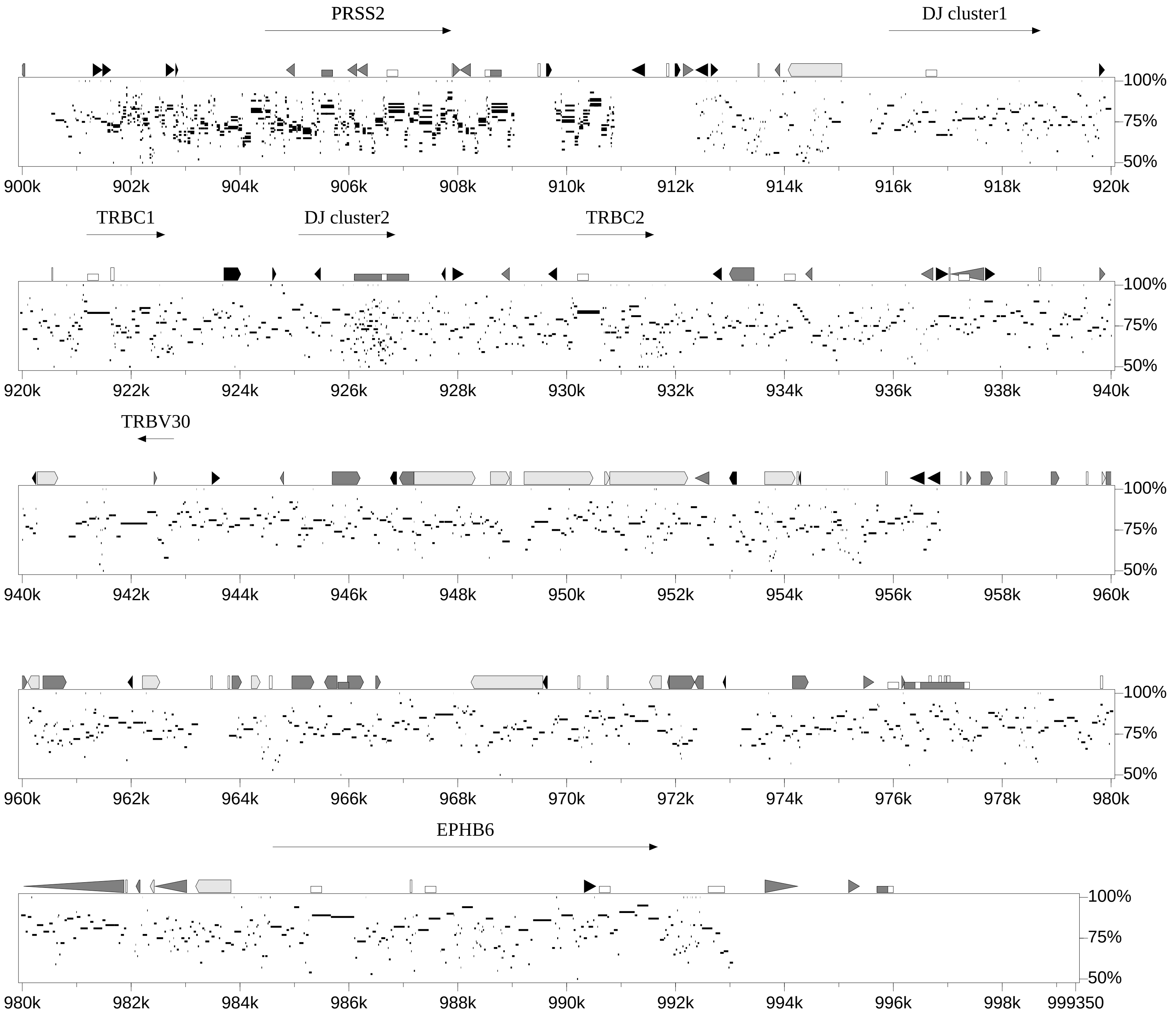

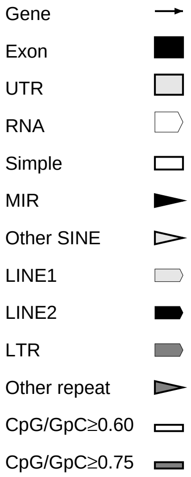

Supplement: Supplementary file 1 [file animals-14-02817-s001.zip › Figure S3.pdf]
