# Supplementary material for: A Comprehensive Analysis of the Genomic and Expressed Repertoire of the T-Cell Receptor Beta Chain in Equus caballus
Source: Animals (Basel). 2024 Sep 29;14(19):2817. doi: 10.3390/ani14192817 (PMC11475548; doi:10.3390/ani14192817)
Supplement: Supplementary file 1 [file animals-14-02817-s001.zip › Table S1.pdf]

Table S1. Description of the TRB genes in the *Equus caballus* chromosome 4 genome assembly (NCBI Reference Sequence NC_009147). The position of all genes and their classification and functionality are reported. The position and functionality of the TRY genes is also reported.

Gene classification	Functionality	Position[a]
TRBV1	F	95092983-95093616
TRY1	F	95103788-95110036
TRY2	F	95115768-95121952
TRY3	P	95129139-95133471
TRY4	F	95142197-95147248
TRY5	F	95167586-95172510
TRY6	F	95180192-95184292
TRBV4-1	P[1]	95192580-95193024
TRBV2-1	P[2]	95194064-95194531
TRBV3-1	F	95204414-95204873
TRBV4-2	P[3]	95209501-95209939
TRBV2-2	F	95212105-95212572
TRBV5-1	P[4]	95218200-95218658
TRBV5-2	F	95221869-95222345
TRBV6-1	F[b]	95226941-95227384
TRBV7-1	P[5]	95228629-95229074
TRBV8-1	P[3]	95231724-95232192
TRBV5-3	P[6]	95236546-95236989
TRBV7-2	F	95242450-95242887
TRBV13-1	P[3]	95248816-95249293
TRBV10-1	F	95252021-95252472
TRBV11-1	P[3]	95256259-95256703
TRBV12-1	ORF[7]	95262123-95262554
TRBV12-2	F	95269089-95269531
TRBV14-1	P[8]	95274515-95274940
TRBV15-1	F	95276367-95276839
TRBV20-1	P[9]	95278728-95279200
TRBV3-2	F	95285336-95285795
TRBV4-3	P[10]	95289982-95290431
TRBV5-4	ORF[11]	95305113-95305552
TRBV5-5	P[3]	95310135-95310607
TRBV6-2	F	95314159-95314603
TRBV7-3	P[10]	95320515-95320958
TRBV8-2	P[12]	95323596-95324064
TRBV5-6	P[3]	95330543-95330983
TRBV7-4	P[3]	95336447-95336880
TRBV13-2	P[1]	95342570-95343031
TRBV10-2	P[4]	95347265-95347716
TRBV11-2	P[13]	95349722-95350724
TRBV12-3	P[14]	95362622-95363062
TRBV5-7	F	95371586-95372045
TRBV6-3	F[b]	95376830-95377274
TRBV7-5	P[13]	95386155-95386894
TRBV8-3	P[15]	95389488-95389958
TRBV8-4	ORF[16]	95394554-95395021

TRBV5-8	P[17]	95399369-95399838
TRBV5-9	P[3]	95404424-95404893
TRBV8-5	ORF[16]	95409536-95410005
TRBV5-10	P[3]	95414595-95415063
TRBV13-3	P[4]	95420837-95421313
TRBV10-3	P[4]	95426062-95426513
TRBV11-3	P[3]	95433638-95434084
TRBV12-4	P[18]	95435583-95436029
TRBV14-2	F	95445414-95445854
TRBV15-2	F	95447276-95447745
TRBV16-1	P[3]	95452648-95453101
TRBV17-1	P[19]	95455679-95456112
TRBV18-1	P[3]	95463068-95463686
TRBV5-11	P[13]	95481664-95482194
TRBV5-12	F	95485163-95485639
TRBV6-4	F	95489913-95490355
TRBV7-6	P[3]	95499330-95499772
TRBV16-2	P[3]	95501621-95502070
TRBV17-2	P[3]	95504379-95504816
TRBV12-5	F	95508274-95508705
TRBV5-13	F	95513828-95514287
TRBV5-14	P[3]	95517538-95518001
TRBV7-7	F	95528757-95529194
TRBV12-6	P[4]	95539518-95539949
TRBV14-3	F	95545208-95545648
TRBV15-3	P[4]	95547063-95547531
TRBV16-3	P[3]	95551667-95552117
TRBV17-3	P[17]	95554503-95554935
TRBV18-2	P[3]	95562816-95563454
TRBV19-1	P[3]	95566726-95567197
TRBV20-2	P[20]	95573624-95574073
TRBV4-4	P[17]	95579891-95580336
TRBV2-3	P[21]	95581397-95581848
TRBV3-3	F	95591602-95592060
TRBV4-5	P[22]	95596480-95596936
TRBV5-15	F	95611658-95612117
TRBV5-16	P[14]	95615372-95615838
TRBV6-5	F	95620360-95620804
TRBV7-8	P[3]	95624099-95624534
TRBV8-6	P[3]	95627413-95627885
TRBV5-17	P[15]	95632089-95632534
TRBV7-9	F	95637949-95638404
TRBV14-4	F	95663908-95664342
TRBV15-4	P[19]	95666033-95666495
TRBV16-4	F	95670538-95670991
TRBV17-4	P[17]	95673679-95674112
TRBV12-7	F	95677332-95677763
TRBV5-18	P[3]	95689385-95689845
TRBV5-19	F	95693354-95693830
TRBV6-6	F[b]	95698358-95698802
TRBV7-10	P[23]	95705751-95706195

TRBV8-7	P[4]	95708862-95709333
TRBV5-20	P[17]	95713940-95714373
TRBV7-11	F	95719705-95720147
TRBV13-4	F	95725919-95726398
TRBV10-4	P[3]	95730725-95731181
TRBV11-4	F	95735196-95735643
TRBV15-5	F	95743566-95744035
TRBV16-5	F	95748091-95748544
TRBV17-5	P[17]	95751169-95751601
TRBV18-3	P[21]	95758843-95759480
TRBV19-2	P[24]	95761295-95761591
TRBV20-3	F	95766995-95767464
TRBV20-4	P[13]	95773014-95773945
TRBV21-1	F	95784309-95784770
TRBV22-1	P[6]	95788094-95788542
TRBV23-1	P[3]	95792762-95793220
TRBV21-2	F	95803991-95804449
TRBV22-2	ORF[25]	95808831-95809294
TRBV23-2	P[3]	95813504-95813968
TRBV19-3	P[19]	95820587-95821060
TRBV20-5	P[26]	95826203-95826932
TRBV21-3	F	95833795-95834253
TRBV23-3	P[27]	95841061-95841529
TRBV18-4	P[14]	95846363-95847003
TRBV18-5	P[19]	95849720-95850335
TRBV19-4	P[3]	95852629-95853103
TRBV20-6	P[28]	95857281-95857951
TRBV18-6	P[29]	95861666-95862283
TRBV19-5	P[14]	95864607-95865075
TRBV20-7	P[30]	95870026-95870498
TRBV18-7	P[31]	95874116-95874751
TRBV19-6	P[3]	95877158-95877623
TRBV20-8	P[12]	95883074-95883541
TRBV21-4	F	95894546-95895007
TRBV23-4	P[32]	95899911-95900380
TRBV24	P[33]	95907213-95907683
TRBV25	F[b]	95913185-95913654
TRBV26-1	P[17]	95923601-95924091
TRBV27-1	F	95931449-95931918
TRBV26-2	P[17]	95935321-95935809
TRBV27-2	F	95943790-95944257
TRBV28-1	F	95955846-95956290
TRBV28-2	F	95962049-95962493
TRBV28-3	F	95969045-95969509
TRBV29	F	95975338-95975957
TRY7	F	95985525-95988943
TRBD1	F	95996985-95996996
TRBJ1-1	F	95997629-95997676
TRBJ1-2	F	95997764-95997811
TRBJ1-3	F	95998386-95998435
TRBJ1-4	F	95998989-95999039

TRBJ1-5	F	95999261-95999310
TRBJ1-6	F	95999720-95999772
TRBC1	F	96002247-96003690
TRBD2	F	96006141-96006157
TRBJ2-1	F	96006810-96006859
TRBJ2-2	F	96007008-96007058
TRBJ2-3	F	96007271-96007319
TRBJ2-4	F	96007418-96007466
TRBJ2-5	F	96007527-96007575
TRBJ2-6	F	96007651-96007702
TRBJ2-7	F	96007873-96007919
TRBC2	F	96011247-96012670
TRBV30	P[3]	96023183-96023852

[a] L-PART1/ V-exon for TRBV genes;

[b] STOP CODON at position 108 (last 3' codon of germline CDR3-IMGT) may disappear during rearrangements

[1] frameshift in L-PART1, frameshift in V-exon
[2] STOP-CODON in L-PART1 and in V-exon, no TRP41
[3] frameshift in V-exon
[4] STOP CODON in V-exon
[5] STOP CODONs, frameshift in V-exon
[6] STOP CODON in V exon, noncanonical ACCEPTOR-SPLICE
[7] no CYS23
[8] del in L-PART1, STOP-CODON in V-exon
[9] noncanonical L-PART1 and DONOR-SPLICE
[10] noncanonical L-PART1, STOP CODONs in V exon
[11] del in V-exon
[1 2] frameshift in L-PART1
[13] Insertion of genomic sequence in coding region
[14] no INIT-CODON, frameshift in V-exon
[15] no INIT-CODON
[16] noncanonical ACCEPTOR-SPLICE
[17] noncanonical DONOR-SPLICE, frameshift in V-exon
[18] no INIT-CODON, noncanonical ACCEPTOR-SPLICE, STOP CODONs in V exon
[19] noncanonical ACCEPTOR-SPLICE, frameshift in V-exon
[20] noncanonical L-PART1 and DONOR and ACCEPTOR-SPLICE, frameshift in V-exon
[21] STOP CODONs in V-exon
[22] STOP CODONs in L-PART1 and in V-exon, noncanonical DONOR-SPLICE
[23] noncanonical L-PART1 and ACCEPTOR -SPLICE, frameshift in V-exon
[24] no L-PART1
[25] no TRP41 and CYS23
[26] no INIT-CODON, noncanonical ACCEPTOR-SPLICE
[27] del in L-PART1, frameshift in V-exon, noncanonical RS
[28] noncanonical L-PART1 and DONOR and ACCEPTOR-SPLICE
[29] noncanonical ACCEPTOR-SPLICE, STOP CODONS in V exon
[30] noncanonical DONOR-SPLICE
[31] STOP CODONs in L-PART1, noncanonical ACCEPTOR-SPLICE, frameshift in V-exon
[32] del in L-PART1
[33] STOP CODON in V-exon, no CYS104
