# Supplementary material for: A Comprehensive Analysis of the Genomic and Expressed Repertoire of the T-Cell Receptor Beta Chain in Equus caballus
Source: Animals (Basel). 2024 Sep 29;14(19):2817. doi: 10.3390/ani14192817 (PMC11475548; doi:10.3390/ani14192817)
Supplement: Supplementary file 1 [file animals-14-02817-s001.zip › Table S2.pdf]

Table S2. Output file of the program RepeatMasker. Summary of the repeat content of horse TRBV-cluster is shown. The GC levels are also indicated.

```
==================================================
file name: Horse TRBV-cluster
total length:     907877 bp
GC level:         41.62 %
bases masked:     275499 bp (30.35 %)
==================================================
               number of      length   percentage
               elements*    occupied  of sequence
--------------------------------------------------
SINEs:                97       15105 bp    1.66 %
      Alu/B1           0           0 bp    0.00 %
      MIRs            97       15105 bp    1.66 %

LINEs:               300      179503 bp   19.77 %
      LINE1          213      155529 bp   17.13 %
      LINE2           76       22007 bp    2.42 %
      L3/CR1          10        1631 bp    0.18 %
      RTE              1         336 bp    0.04 %

LTR elements:        150       55197 bp    6.08 %
      ERVL            65       25142 bp    2.77 %
      ERVL-MaLRs      51       18031 bp    1.99 %
      ERV_classI      27       11215 bp    1.24 %
      ERV_classII      4         334 bp    0.04 %

DNA elements:         90       16347 bp    1.80 %
      hAT-Charlie     41        8468 bp    0.93 %
      TcMar-Tigger     5         891 bp    0.10 %

Unclassified:          0           0 bp    0.00 %

Total interspersed repeats:   266152 bp   29.32 %


Small RNA:             4         263 bp    0.03 %

Satellites:            2         183 bp    0.02 %
Simple repeats:      188        7060 bp    0.78 %
Low complexity:       39        1895 bp    0.21 %
==================================================
*most repeats fragmented by insertions or deletions have
been counted as one element
```
